# New-Onset Catatonia and Delirium in a COVID-Positive Patient

**DOI:** 10.7759/cureus.18422

**Published:** 2021-10-01

**Authors:** Gagan Kaur, Zeba Khavarian, Sayeda A Basith, Farzana Faruki, Charles Mormando

**Affiliations:** 1 Medicine and Surgery, Sri Guru Ram Das Institute of Medical Sciences and Research, Amritsar, IND; 2 Family Medicine, Saba University School of Medicine, The Bottom, NLD; 3 Psychiatry and Behavioral Sciences, Medical University of the Americas, Charlestown, KNA; 4 Internal Medicine, California Institute of Behavioral Neurosciences and Psychology, Fairfield, USA; 5 Psychiatry, Penn State University College of Medicine, Milton S. Hershey Medical Center, Hershey, USA

**Keywords:** electroconvulsive, ect, psychotropics, covid-19, delirium, catatonia

## Abstract

Coronavirus disease (COVID-19) is a strain of coronavirus family, which was initially found in China in late 2019 and subsequently spread to rest of the world. COVID-19 has led to physical and mental health complications since its onset. In addition to the pandemic-associated social stresses, biological complications include direct viral encephalitis, autoimmune-mediated responses, medication side effects, hypoxic brain injury, and delirium, which can collectively cause varied presentations of neuropsychiatric symptoms. Neuropsychiatric complications have been reported in the acute stages of COVID-19 and post-infection period. Here we report our experience treating a patient who initially presented with a severe depressive episode and subsequently developed catatonia and delirium following hospital-acquired COVID-19 infection.

## Introduction

Coronavirus disease (COVID-19) is a strain of the coronavirus family that was initially identified in Wuhan, China in late 2019, and subsequently quickly spread across the globe. The pandemic has led to worsening of existing mental health conditions and new onset of psychiatric illnesses in the context of increased stressors in adults and children alike [1,2]. The COVID-19 pandemic has been a cause of significant psychological stress for most individuals, and social factors such as isolation, anxiety, and financial difficulties have been implicated in playing a role in the development of various neuropsychiatric complications [3]. In addition to these pandemic-associated social stresses, biological complications include direct viral encephalitis, autoimmune-mediated responses, medication side effects, hypoxic brain injury, and delirium, which can collectively cause varied presentations of neuropsychiatric symptoms [4]. Neuropsychiatric complications have been reported in the acute stages of COVID-19 and post-infection period. Of particular interest, there are reports of patients developing catatonia [5].

Catatonia is a distinct neuropsychiatric syndrome characterized by motor and behavior dysfunction. The syndrome is becoming more recognized clinically and in ongoing research; it occurs with psychiatric, metabolic, and neurologic conditions. The Bush-Francis Catatonia Rating Scale (BFCRS) is the most widely utilized rating scale in research and clinical practice [6]. Treatment with benzodiazepines or electroconvulsive therapy (ECT) usually produces dramatic and rapid response, although systematic, randomized trials are lacking. The role of antipsychotic agents in treatment is controversial as they may worsen the syndrome. An important unresolved clinical question is the diagnosis and treatment of catatonia in the setting of delirium [6].

There is limited data on the treatment of catatonia and co-morbid delirium in the setting of COVID-19 infection. Here we report our experience treating a patient who initially presented with a severe depressive episode and subsequently developed catatonia and delirium following hospital-acquired COVID-19 infection. We discuss the possible neurobiological mechanisms and some of the interactions between antiretroviral therapies and psychotropic medications and anesthetic drugs utilized during ECT.

## Case presentation

A 59-year-old single and unemployed Caucasian male, with a history of bipolar-spectrum illness, was psychiatrically hospitalized for treatment of severe depression with active suicidal ideation in the setting of various psychosocial stressors. Initial examination was notable for pronounced psychomotor retardation, prolonged speech latency, and flat affect. There was worsening of depressed mood over the prior two weeks that was associated with poor appetite, decreased energy levels, difficulty sustaining attention, and severe insomnia. Past medical history included well-controlled hypertension and hyperlipidemia, and a past psychiatric history notable for unspecified bipolar disorder. Known previous medication trials included valproic acid and lorazepam. He was a prior tobacco smoker with no other substance use history. Medications at admission were continued and included valproic acid 250 mg in the morning and 500 mg at night, atorvastatin 10 mg daily, lisinopril 40 mg daily, and amlodipine 2.5 mg daily. He received Moderna two-dose COVID vaccination series approximately four months prior to admission. All baseline labs were within normal limits, valproic acid level on admission was noted to be within normal limits, and COVID polymerase chain reaction (PCR) testing was negative on admission.

The patient was started on mirtazapine 15 mg at bedtime upon admission and was gradually titrated to 45 mg dose. Within seven days of hospitalization there was an abrupt onset of catatonia followed by delirium. There was partial mutism, severe withdrawal (no oral intake for days with 20-pound weight loss within a week), stupor, staring, catalepsy, and negativism. Initial BFCRS was 12. He was clearly disoriented, unable to sustain attention, and there were fluctuations in level of consciousness. Repeat PCR testing was performed and was positive for COVID-19. Complete blood count and comprehensive metabolic panel were within normal limits at that time. However, dehydration, malnutrition, hypotension, and acute kidney failure ensued and he was transferred to a local medical hospital given rapid decompensation. He was treated symptomatically and received intravenous fluids. The patient never developed the typical respiratory symptoms of COVID-19, which was possibly secondary to his vaccination status. He returned to psychiatry once his vital signs and kidney function normalized.

Oral lorazepam was started and titrated to 2 mg three times a day, to target catatonia. This was done judiciously in the setting of delirium and orthostatic hypotension due to inadequate intake. He was unable to tolerate lorazepam beyond 6 mg daily and titration was stopped. Court-ordered ECT was obtained and started once he was medically cleared and quarantine was over. He received six brief-pulse bitemporal treatments with excellent response and tolerability as the catatonia and delirium resolved. Methohexital and etomidate were used for general anesthesia. The patient was discharged to outpatient care with psychiatry and therapy follow-up appointments in the community.

## Discussion

This case is particularly interesting because it appears that catatonia may have been the result of new-onset COVID infection. This patient did not have a previous history of catatonia and PCR COVID testing was negative on admission. He developed catatonia and delirium within a week of hospitalization and the second PCR test at that time was positive. It also emphasizes the need for maintaining adequate masking even in the patients who have been vaccinated previously.

COVID-19 infection may result in long-term effects on physical and neuropsychiatric health [7]. The acute effects of COVID-19 on cognitive and behavioral function in some cases appear to be similar to viral encephalitis. The neuropsychiatric manifestations of COVID-19 may include delirium, depression, anxiety, insomnia, agitation, and catatonia [8]. Catatonia can present in COVID-19 patients without prior history of neuropsychiatric disorders [5]. There are several theoretical models of the underlying mechanism of abnormal psychomotor behaviors observed in catatonia. However, no conclusive genetic, pathological, neurochemical, or structural mechanisms have been elucidated. The model of catatonia as a syndrome of motor impairment contains the most neuroscientific data, and focuses primarily on dopamine and gaba-aminobutyric acid, which may be disrupted by COVID infection [3,5].

Coronavirus is thought to enter the central nervous system through the cribriform plate or by hematogenous spread and binds to glycoproteins on the surface of angiotensin-converting enzyme. It then enters through the olfactory bulb to replicate in neurons [8]. Post-mortem examinations of COVID-19 patients have shown cerebral inflammation from an overwhelming cytokine inflammatory response [9,10]. Cytokine elevation and glial activation increase the level of amino acids like glutamate, which reduces neurotransmitter levels and induces hypoxic injury by way of increased coagulation, thus causing neuronal loss [5].

Several psychotropic medications may interact with antiretroviral therapy used to treat the symptoms of COVID-19. Remdesivir is an antiretroviral that is currently used in the treatment of COVID-19 and interacts with concomitant psychiatric medications such as thioridazine, carbamazepine, phenytoin, phenobarbital, and primidone. These combinations require close monitoring as it can decrease the serum levels of remdesivir [11]. Other medications like ritonavir and lopinavir are also used in the treatment of COVID-19. These medications should be used with caution in combination with fluoxetine as this can precipitate serotonin toxicity. Ritonavir should not be administered with duloxetine or trazodone as it increases the serum levels of these drugs and should be given with caution along with antipsychotic medications as it can drastically increase or decrease their levels [11,12]. It is particularly important to ask patients about off-label use of popular non-approved COVID-19 medications such as ivermectin and hydroxychloroquine/chloroquine, as they can have significant interactions with psychotropic medication and COVID-19 medications and can aggravate or precipitate conditions such as delirium [13].

Treatment with benzodiazepines and/or ECT usually results in complete resolution of catatonia [14]. There are no known pharmacokinetic interactions between benzodiazepines or the commonly used anesthetic drugs utilized in ECT and the antiretroviral drugs used in COVID-19 infection. Other medications that may be used in the treatment of catatonia include N-methyl-D-aspartate antagonists and zolpidem [15]. These medications also do not have interactions with the above antiretroviral drugs. However, pharmacodynamic properties must always be taken into account as benzodiazepines and anesthetic drugs may worsen respiratory function in severely ill COVID patients.

In summary, catatonia in the setting of COVID-19 infection responds to benzodiazepines and ECT. Delirium may also be present and should not deter treatment with benzodiazepines or ECT. Although several interactions exist between various psychotropic medications and antiretrovirals, benzodiazepines and the anesthetic drugs utilized during ECT appear to be safe in comorbid COVID-19 infection. 

## Conclusions

COVID-19 pandemic has created huge crises worldwide both in terms of physical and mental health. Management of patients in inpatient psychiatric units has posed a huge challenge in patients who either develop COVID infections during the hospitalizations or those who develop neuropsychiatric sequelae as a result of COVID infection. A particularly challenging scenario is management of catatonia and delirium in COVID-positive patients. Catatonia in the setting of COVID-19 infection responds well to benzodiazepines and ECT. Although several interactions exist between various psychotropic medications and antiretrovirals that are being used to treat COVID infections, benzodiazepines and the anesthetic drugs utilized during ECT appear to be generally safe.
